# The environmentally impacts of digital health

**DOI:** 10.1177/20552076211033421

**Published:** 2021-08-10

**Authors:** Maddy Thompson

**Affiliations:** GGE, 4212 Keele University, Keele, UK

**Keywords:** Digital health, environment, global health, climate change

## Abstract

Digital health interventions are widely celebrated due to their low-cost nature and ability to provide tailored person-centred care in communities worldwide. As coronavirus disease-19 has rapidly accelerated their growth and reach, interest in global ethical questions surrounding digital health is growing. However, the global environmental implications of digital health have been overlooked. This commentary draws attention to the environmental impacts of digital health devices and communication networks, as well as the data produced by digital health activities. Unless serious attention is paid to greening digital health practices, the rise of digital health will significantly contribute to environmental change, and thus create outcomes of ill-health.

Digital health interventions are widely celebrated due to their low-cost nature and ability to provide tailored person-centred care in communities worldwide. As coronavirus disease-19 has rapidly accelerated their growth and reach, interest in global ethical questions surrounding digital *health is rising*. For example, the 2020 Riyadh Declaration on Digital Health provides priorities and recommendations for the global health community, raising ethical issues of data sharing, security, privacy and misinformation.^1^ However, in addressing such issues the global environmental implications of digital health have been overlooked.

Considering climate change disproportionately affects the global south^2^ and global information and communication technology emissions already account for around 3.5% of global carbon emissions,^3^ the omission of environmental factors from digital health debates is significant. There is a pressing need to attend to the environmental impacts of digital health, understanding that in the long-term it can produce outcomes of ill-health for the world's poorest and most vulnerable communities. There are three key areas of interest in this regard – devices, data and communication networks.

First, the production and disposal of wearable technologies, robotics and devices used to facilitate health pi (smartphones, tablets, laptops, etc.) cause environmental degradation. Raw materials required to produce these technologies – iron, aluminium, gold, mercury, cyanide, etc. – require large mining operations, that are largely located in the global south. Spillages of toxic waste, ecosystem destruction and land-use change create significant environmental degradation, while workers may be exposed to toxins.^4^ The carbon required to produce electronic devices is vast, and their energy consumption is around 8% of total global consumption.^3^ The e-waste generated by the disposal of electronic technologies similarly has devastating environmental implications. Only between 10% and 40% of electronic devices are recycled, the rest sent to landfills, releasing toxic chemicals into the environment.^5^ Globally, recycled e-waste is mainly sent to the global south. Inadequate resources to effectively handle e-waste leads to pollution of local environments, creating significant health risks.^6^ The expansion of digital health will increase demand for devices, contributing to the environmental burden of electronics. Serious steps including green mining,^4^ investment into electronic recycling, and supply chain efficiency initiatives such as those undertaken in the Nordic region must be taken to ensure devices are produced and disposed of ethically.

Second, digital health interventions increase the collection and storage of health data. From electronic health records, biometric data collected by wearable technologies and mHealth, to online health searches, digital health produces vast amounts of digital data. Health data accounts for around 30% of the world's total data.^7^ Storing data requires large servers which use vast amounts of electricity to run and keep cool, particularly when data is saved on cloud services. Sending, copying and safely storing data on clouds takes approximately one million times more energy than saving direct to devices.^8^ Furthermore, increasingly big data is used to drive artificial intelligence (AI) and blockchain health technologies. To reduce the environmental impacts of data centres, green cloud computing, the reduction of health data and the use of environmentally conscious computational technologies such as tinyML and compact AI must be key priorities.

Third, are the digital health communications infrastructures that facilitate digital health, including telehealth call centres. Limited research on telehealth hypothesizes that in rural settings telehealth lowers carbon emissions due to reduced transport.^9^ Such gains are likely to be minimal, particularly where public transport is the alternative, but are nonetheless important. More pressing is the large-scale telehealth operations housed in call centres. As with data centres, telecommunications centres require vast amounts of energy to power and cool technology, further contributing to carbon emissions.^3^ In the Philippines, home to a large hub of international telehealth operators, Green Information Technologies are used to reduce the environmental costs associated with communication networks.^10^ Such practices must become commonplace.

Digital health may have the potential to alleviate global health inequalities in the short term, but moving forward there is a need to consider its long-term environmental sustainability. There must be an imperative to make environmental considerations a key priority when developing and implementing digital health. Building on the 2020 Riyadh Declaration on Digital Health,^1^ it is recommended that environmental audits of digital health interventions accounting for environmental impacts of devices, data and computation, and telehealth centres are carried out. Green Information Technologies and ethical sourcing and disposal of devices should be adopted where possible. However, there is also a need to lobby the digital technology field more broadly to ensure sustainable practices become standard, recognising that many digital health technologies result from design and commercialisation decisions beyond the field of health. Ensuring environmental regulations are included in national and global health policy is thus essential to ensure big tech companies are incentivised to invest in more environmentally sustainable technologies. Without such steps, we risk that digital health will lead to additional global health burdens, creating ill-health among the world's most vulnerable.
