# Erratum to: The environmental impacts of digital health

**DOI:** 10.1177/20552076211045559

**Published:** 2021-10-07

Thompson M. “The Environmental Impacts of Digital Health”. *DIGITAL HEALTH*. DOI: 10.1177/20552076211033421

The **Environmentally** within the article title is a publishing error; the correct title is ‘The Environmental Impacts of Digital Health’.

